# Editorial: The potential of transferrin as a drug target and drug delivery system

**DOI:** 10.3389/fphar.2025.1584190

**Published:** 2025-03-14

**Authors:** Patricia Carrilho, Sidharth Chopra, Mallikarjuna Rao Pichika, Robert E. Fleming, Nermi L. Parrow

**Affiliations:** ^1^ Nephrology Department, Hospital Professor Doutor Fernando Fonseca EPE, Amadora, Portugal; ^2^ Department of Molecular Microbiology and Immunology, CSIR-Central Drug Research Institute, Lucknow, India; ^3^ School of Pharmacy, Newcastle University Medicine Malaysia, Johor Bahru, Malaysia; ^4^ Department of Pediatrics, Saint Louis University School of Medicine, Saint Louis, MO, United States

**Keywords:** transferrin, hepcidin, erythropoiesis, targeted drug delivery, transferrin receptor (TfR)

Transferrin (Tf), the major iron-binding protein in plasma, appears to have both cargo and signaling functions. It has two homologous lobes, N and C, each of which can bind one iron molecule. Clefts in these lobes close upon binding iron (Luck and Mason, 2012), resulting in a conformational shift that alters the affinity for transferrin receptors and may have additional consequences for protein-protein interactions. The ability of each lobe to independently bind an iron atom results in four circulating species of Tf: apoTf, monoferric N, monoferric C, and holoTf. The monoferric forms of transferrin influence erythropoietin sensitivity and iron homeostasis (Parrow et al., 2019). Thus, targeting them may have therapeutic potential in diseases characterized by erythropoietin resistance or dysfunctional hepcidin levels.

Transferrin has two receptors: transferrin receptor 1 (TFR1) and transferrin receptor 2 (TFR2). TFR1 is ubiquitously expressed, including in the brain endothelium (Roberts et al., 1993), and primarily serves to deliver iron from transferrin to cells. At serum pH, iron-loaded Tf binds TFR1 with high affinity and the complex undergoes endocytosis. A decrease in endosomal pH promotes the release of iron from Tf and the apo-Tf-TfR1 complex is recycled to the cell surface where apoTf is released (Steere et al., 2012). TFR1 is upregulated in highly proliferative cells (Zhang et al., 2024) and in response to iron deficiency (Tong et al., 2002; O'Donnell et al., 2006; Meyron-Holtz et al., 2004).

TFR2 is thought to primarily serve as a signaling receptor. In mice (Fleming et al., 2002) and humans (Camaschella et al., 2000), loss of TFR2 results in hereditary hemochromatosis, a disorder characterized by excessive iron absorption secondary to inappropriately low hepcidin levels.

This Research Topic focuses on Tf as a therapeutic target and a drug delivery system.

Li et al. present a review of the potential therapeutic applications of Tf (*Pathophysiological aspects of transferrin-A potential nano-based drug delivery signaling molecule in therapeutic target for varied diseases*). They provide an overview of the use of TF as a therapeutic agent in various clinical conditions. One of these is transferrin replacement therapy in the rare transferrin deficiency disease, atransferrinemia. Another is the use of transferrin therapies, such as the administration of ApoTf, to neutralize free iron in ischemia reperfusion injury models. This review also covers an array of studies where chemotherapeutics or imaging agents are delivered via transferrin-conjugated moieties or are otherwise directed to TfR1 by an antibody or peptide. These approaches leverage the upregulation of TfR1 that characterizes proliferating tumor cells.

He et al. also present studies investigating transferrin-conjugated therapies or TfR1-targeting in the context of reviewing the potential for natural products to alleviate chemotherapy-related cognitive impairment (*Natural products for the treatment of chemotherapy-related cognitive impairment and prospects of nose-to-brain drug delivery*). One such study indicates that targeting nanocarrier-wrapped ginsenoside RG1 (an active constituent of ginseng) to TfR1 increased penetration of the blood brain barrier and decreased brain infarct volume in a cerebral ischemia model (Shen et al., 2019). He et al. also propose nose-to-brain delivery as a mechanism to bypass the blood-brain barrier. Proof of principle is provided by at least one study that generated and investigated transferrin-conjugated chitosan nanoparticles (Gabold et al., 2023). Nanoparticles with the highest surface expression of transferrin had the highest uptake in a human nasal epithelial cell line and transferrin-conjugated nanoparticles passed more quickly through an epithelial layer to glioblastoma cells in co-culture model systems.

In a specific study of transferrin as a delivery system, Alrouji et al. present a primary investigation of the binding mechanism of capsaicin with human transferrin (*Evaluation of binding mechanism of dietary phytochemical, capsaicin, with human transferrin: targeting neurodegenerative diseases therapeutics*). Capsaicin is the primary component of chili pepper. Recent studies suggest dietary capsaicin may have neuroprotective properties, with specific therapeutic potential for Alzheimer’s disease (Wang et al., 2020; Xu et al., 2017). Transferrin provides a potential means to deliver capsaicin across the blood-brain barrier via TfR1. Using a combination of approaches, their studies indicate that capsaicin binds transferrin in the iron-binding pocket without significant structural alterations. They report a binding constant of 3.99 × 10^6^ M^-1^, indicating that transferrin has a considerably lower affinity for capsaicin compared to Fe^3+^ (Aisen et al., 1978). *In vivo* studies comparing the efficacy of transferrin-mediated capsaicin delivery to dietary capsaicin in mouse models of disease will be useful in evaluating the feasibility of this approach.

Ren et al. present an umbrella review of meta-analyses of the effects of the prolyl hydroxylase inhibitors (PHI) on anemia in chronic kidney disease (*Efficacy and safety of hypoxia-inducible factor-prolyl hydroxylase inhibitor treatment for anemia in chronic kidney disease: an umbrella review of meta-analyses*). This class of drugs inhibits the oxygen-dependent prolyl hydroxylases responsible for degradation of hypoxia inducible factor (HIF) (Haase, 2021). HIF controls the myriad responses to hypoxia (Semenza, 2012), and transferrin itself is upregulated in response to hypoxia (Li et al., 2022). Their meta-analysis confirms an increase of ∼ 1 g of hemoglobin per deciliter in studies of PHIs. As expected, the increase is more pronounced compared to placebo than to erythropoiesis-stimulating agents (ESAs). Treatment with PHIs decreases levels of the hepatic iron-regulatory hormone hepcidin, also demonstrating a stronger effect compared to placebo than to ESAs. Surprisingly, 7 of 11 studies analyzed did not show a significant difference in serum iron concentration compared to ESA or placebo. There was, however, evidence of increased total iron-binding capacity, a surrogate measure of transferrin, and transferrin level itself. A corresponding decrease in transferrin saturation was also observed. Based on the demonstrated capacity of lobe occupancy of transferrin to influence erythropoietin responsiveness and iron homeostasis, it is tempting to speculate that the upregulation of transferrin by the prolyl hydroxylase inhibitors may have previously unrecognized effects on the distribution of iron-bound transferrin species that contribute to the efficacy of these therapeutics in the anemia of chronic kidney disease.

In total, this Research Topic provides exciting evidence that transferrin has therapeutic utility in a variety of diseases. Additionally, targeting TfR1 may provide specific mechanisms for delivering chemotherapeutics and imaging modalities to rapidly proliferating cells, as well as bypassing the blood-brain barrier. We look forward to the continued development of drugs based on transferrin.

## References

[B1] Aisen P. Leibman A. Zweier J. (1978). Stoichiometric and site characteristics of the binding of iron to human transferrin. J. Biol. Chem. 253, 1930–1937. 10.1016/s0021-9258(19)62337-9 204636

[B2] Camaschella C. Roetto A. Cali A. De Gobbi M. Garozzo G. Carella M. (2000). The gene TFR2 is mutated in a new type of haemochromatosis mapping to 7q22. Nat. Genet. 25, 14–15. 10.1038/75534 10802645

[B3] Fleming R. E. Ahmann J. R. Migas M. C. Waheed A. Koeffler H. P. Kawabata H. (2002). Targeted mutagenesis of the murine transferrin receptor-2 gene produces hemochromatosis. Proc. Natl. Acad. Sci. U. S. A. 99, 10653–10658. 10.1073/pnas.162360699 12134060 PMC125003

[B4] Gabold B. Adams F. Brameyer S. Jung K. Ried C. L. Merdan T. (2023). Transferrin-modified chitosan nanoparticles for targeted nose-to-brain delivery of proteins. Drug Deliv. Transl. Res. 13, 822–838. 10.1007/s13346-022-01245-z 36207657 PMC9892103

[B5] Haase V. H. (2011). Hypoxia-inducible factor-prolyl hydroxylase inhibitors in the treatment of anemia of chronic kidney disease. Kidney Int. Suppl. 11, 8–25. 10.1016/j.kisu.2020.12.002 PMC7983025 33777492

[B6] Li M. Tang X. Liao Z. Shen C. Cheng R. Fang M. (2022). Hypoxia and low temperature upregulate transferrin to induce hypercoagulability at high altitude. Blood 140, 2063–2075. 10.1182/blood.2022016410 36040436 PMC10653030

[B7] Luck A. N. Mason A. B. (2012). Transferrin-mediated cellular iron delivery. Curr. Top. Membr. 69, 3–35. 10.1016/B978-0-12-394390-3.00001-X 23046645 PMC4479283

[B8] Meyron-Holtz E. G. Ghosh M. C. Iwai K. Lavaute T. Brazzolotto X. Berger U. V. (2004). Genetic ablations of iron regulatory proteins 1 and 2 reveal why iron regulatory protein 2 dominates iron homeostasis. EMBO J. 23, 386–395. 10.1038/sj.emboj.7600041 14726953 PMC1271751

[B9] O'Donnell K. A. Yu D. Zeller K. I. Kim J. W. Racke F. Thomas-Tikhonenko A. (2006). Activation of transferrin receptor 1 by c-Myc enhances cellular proliferation and tumorigenesis. Mol. Cell Biol. 26, 2373–2386. 10.1128/MCB.26.6.2373-2386.2006 16508012 PMC1430295

[B10] Parrow N. L. Li Y. Feola M. Guerra A. Casu C. Prasad P. (2019). Lobe specificity of iron binding to transferrin modulates murine erythropoiesis and iron homeostasis. Blood 134, 1373–1384. 10.1182/blood.2018893099 31434707 PMC6839954

[B11] Roberts R. L. Fine R. E. Sandra A. (1993). Receptor-mediated endocytosis of transferrin at the blood-brain barrier. J. Cell Sci. 104 (Pt 2), 521–532. 10.1242/jcs.104.2.521 8505377

[B12] Semenza G. L. (2012). Hypoxia-inducible factors in physiology and medicine. Cell 148, 399–408. 10.1016/j.cell.2012.01.021 22304911 PMC3437543

[B13] Shen J. Zhao Z. Shang W. Liu C. Zhang B. Xu Z. (2019). Fabrication and evaluation a transferrin receptor targeting nano-drug carrier for cerebral infarction treatment. Artif. Cells Nanomed Biotechnol. 47, 192–200. 10.1080/21691401.2018.1548471 30663409

[B14] Steere A. N. Byrne S. L. Chasteen N. D. Mason A. B. (2012). Kinetics of iron release from transferrin bound to the transferrin receptor at endosomal pH. Biochim. Biophys. Acta 1820, 326–333. 10.1016/j.bbagen.2011.06.003 21699959 PMC3253137

[B15] Tong X. Kawabata H. Koeffler H. P. (2002). Iron deficiency can upregulate expression of transferrin receptor at both the mRNA and protein level. Br. J. Haematol. 116, 458–464. 10.1046/j.0007-1048.2001.03289.x 11841452

[B16] Wang J. Sun B. L. Xiang Y. Tian D. Y. Zhu C. Li W. W. (2020). Capsaicin consumption reduces brain amyloid-beta generation and attenuates Alzheimer's disease-type pathology and cognitive deficits in APP/PS1 mice. Transl. Psychiatry 10, 230. 10.1038/s41398-020-00918-y 32661266 PMC7359297

[B17] Xu W. Liu J. Ma D. Yuan G. Lu Y. Yang Y. (2017). Capsaicin reduces Alzheimer-associated tau changes in the hippocampus of type 2 diabetes rats. PLoS One 12, e0172477. 10.1371/journal.pone.0172477 28225806 PMC5321461

[B18] Zhang D. Duque-Jimenez J. Facchinetti F. Brixi G. Rhee K. Feng W. W. (2024). Transferrin receptor targeting chimeras for membrane protein degradation. Nature 638, 787–795. 10.1038/s41586-024-07947-3 39322661 PMC11839386

